# Whole Blood Metabolite Profiles Reflect Changes in Energy Metabolism in Heart Failure

**DOI:** 10.3390/metabo12030216

**Published:** 2022-02-27

**Authors:** Carl Beuchel, Julia Dittrich, Janne Pott, Sylvia Henger, Frank Beutner, Berend Isermann, Markus Loeffler, Joachim Thiery, Uta Ceglarek, Markus Scholz

**Affiliations:** 1 Institute for Medical Informatics, Statistics and Epidemiology, University of Leipzig, 04107 Leipzig, Germany; janne.pott@imise.uni-leipzig.de (J.P.); sylvia.henger@imise.uni-leipzig.de (S.H.); markus.loeffler@imise.uni-leipzig.de (M.L.); 2 Institute of Laboratory Medicine, Clinical Chemistry and Molecular Diagnostics, University Hospital Leipzig, 04103 Leipzig, Germany; julia.dittrich@medizin.uni-leipzig.de (J.D.); berend.isermann@medizin.uni-leipzig.de (B.I.); thiery@med.uni-kiel.de (J.T.); 3 LIFE—Leipzig Research Center for Civilization Diseases, Leipzig University, 04103 Leipzig, Germany; 4 Heart Center Leipzig, 04289 Leipzig, Germany; beutner.le@googlemail.com; 5 Faculty of Medicine, Christian-Albrecht University of Kiel, 24118 Kiel, Germany; 6 IFB AdiposityDiseases, University Hospital Leipzig, 04103 Leipzig, Germany

**Keywords:** cardiovascular disease, coronary artery disease, amino acids, acylcarnitines, gene expression, observational studies, association study, fatty acid oxidation

## Abstract

A variety of atherosclerosis and cardiovascular disease (ASCVD) phenotypes are tightly linked to changes in the cardiac energy metabolism that can lead to a loss of metabolic flexibility and to unfavorable clinical outcomes. We conducted an association analysis of 31 ASCVD phenotypes and 97 whole blood amino acids, acylcarnitines and derived ratios in the LIFE-Adult (*n* = 9646) and LIFE-Heart (*n* = 5860) studies, respectively. In addition to hundreds of significant associations, a total of 62 associations of six phenotypes were found in both studies. Positive associations of various amino acids and a range of acylcarnitines with decreasing cardiovascular health indicate disruptions in mitochondrial, as well as peroxisomal fatty acid oxidation. We complemented our metabolite association analyses with whole blood and peripheral blood mononuclear cell (PBMC) gene-expression analyses of fatty acid oxidation and ketone-body metabolism related genes. This revealed several differential expressions for the heart failure biomarker N-terminal prohormone of brain natriuretic peptide (NT-proBNP) in peripheral blood mononuclear cell (PBMC) gene expression. Finally, we constructed and compared three prediction models of significant stenosis in the LIFE-Heart study using (1) traditional risk factors only, (2) the metabolite panel only and (3) a combined model. Area under the receiver operating characteristic curve (AUC) comparison of these three models shows an improved prediction accuracy for the combined metabolite and classical risk factor model (AUC = 0.78, 95%-CI: 0.76–0.80). In conclusion, we improved our understanding of metabolic implications of ASCVD phenotypes by observing associations with metabolite concentrations and gene expression of the mitochondrial and peroxisomal fatty acid oxidation. Additionally, we demonstrated the predictive potential of the metabolite profile to improve classification of patients with significant stenosis.

## 1. Introduction

The human heart hydrolyzes a total of 6 kg of adenosine tri-phosphate (ATP) per day to maintain contractile function [1]. Reserves that can be mobilized by the myocardium meet the energy demands for about two seconds [2]. Thus, energy production needs to be tightly linked to energy expenditure. This high energy demand is primarily met from mitochondrial fatty acid oxidation (FAO), but the myocardium is characterized as highly flexible in its substrate choice and is able to utilize a variety of substrates, such as lactate, ketone bodies, glucose and amino acids [3]. Most cardiovascular diseases exhibit some form of disturbed cardiac metabolism. Metabolic flexibility is jeopardized in the failing heart as a result of metabolic remodeling occurring under stress. Specifically, under hypertrophy, a decrease in FAO and an increase in glycolysis have been frequently observed [1]. This remodeling is a complex process that leads to a maladaptive spiral and eventually ATP depletion by affecting a multitude of metabolic pathways such as Ca^2+^ homeostasis, creation of reactive oxygen species (ROS) and inflammation [2]. The precise characterization of these disturbances is difficult, with descriptions of the failing heart reaching from “an engine out of fuel” to “an engine flooded with fuel” [3,4].

Analysis of amino acids (AAs) and acylcarnitines (ACs) is common practice in the screening of newborns, but in the recent years, it has also gained attention in characterizing and understanding complex metabolic diseases of adults [5]. Targeted liquid chromatography tandem mass spectrometry is widely applied to quantify levels of known metabolites from dried blood spots (DBS) [6,7]. DBS offer several advantages over the use of other tissues in large-scale screenings of amino acids and acylcarnitines [8,9]. Using different metabolomics platforms, numerous studies have aimed at characterizing various atherosclerosis and cardiovascular disease (ASCVD) phenotypes and establishing metabolic signatures [10]. These signatures often describe an increase in long-chain acylcarnitines with worsening cardiovascular health [11,12]. For instance, Ruiz et al. expanded on the mounting evidence of pathophysiological implications of the energy metabolism for heart failure and described a signature of long-chain acylcarnitines that correlates with reduced left ventricular ejection fraction [13]. Cheng et al. demonstrated the predictive potential of the metabolite profile compared to conventional biomarkers in the diagnosis and prognosis of heart failure [14]. However, these studies were mostly limited to one ASCVD phenotype each, not allowing for comparisons of metabolic profiles across phenotypes. This is especially limiting due to the high heterogeneity of the investigated phenotypes. Additionally, existing studies often fielded a limited sample size, did not correct for confounders and risk factors such as diabetes status, reported unvalidated associations and investigated only a limited metabolic profile including uncharacterized features.

In this study, we aimed to reliably characterize alterations and perturbations of 27 AAs, 35 ACs, free carnitine, the sum of ACs and 34 derived ratios in association with 31 ASCVD phenotypes determined in two large and independent cohorts. Then, we performed an integrative analysis of whole blood and peripheral blood mononuclear cell (PBMC) expression of genes involved in fatty acid oxidation with respect to ASCVD phenotypes. Finally, we aimed to demonstrate the potential of these metabolites to predict significant stenosis (coronary artery disease (CAD) status) by constructing and comparing prediction models using classical risk factors complemented by a metabolite panel.

## 2. Results

### 2.1. Association Analysis of Metabolites and ASCVD Phenotypes

We studied the relationships of metabolic profiles (AAs, ACs and physiological quotients, *n* = 97, see methods, Table S1) and different ASCVD phenotypes (carotid, coronary and peripheral artery disease, vascular stiffness, echocardiographic parameters and heart failure, *n* = 31, Table S2) in two studies, the population-based LIFE-Adult study and LIFE-Heart—a study of patients with suspected or confirmed CAD. For this purpose, we first performed (1) a univariate regression analysis with metabolites as the dependent variable and ASCVD phenotypes as predictors followed by (2) a multivariate regression analysis including 15 previously determined risk factors and confounders as covariates (see methods, Figure S1).

**Figure 1:**
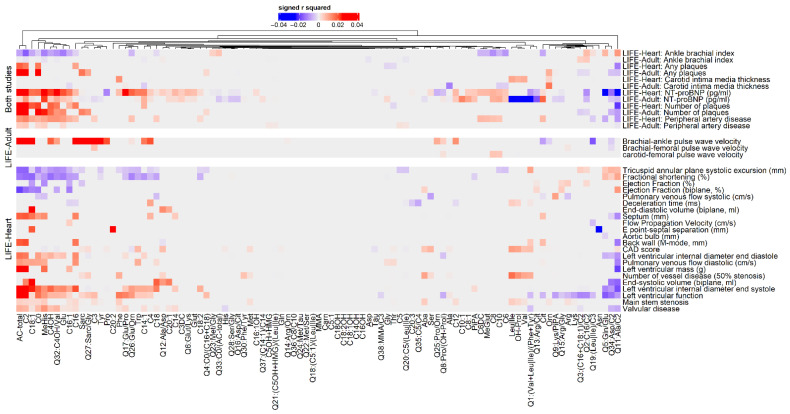
Associations of whole blood metabolites as response variable with ASCVD phenotypes, adjusted for 15 risk factors and confounders in LIFE-Adult and LIFE-Heart. Partial explained variance (partial-r^2^) and direction of effects are displayed for significant associations fulfilling hierarchical FDR < 5% (color indicates significance and direction of effect, intensity of color indicates effect size). Only significant associations are depicted. All statistics are shown in supplemental Table S4. Phenotypes are presented in three groups: “both studies” comprise phenotypes available in both studies, while the other groups are study-specific. Hierarchical clustering based on Euclidean distance using the complete linkage method was applied for metabolites and phenotypes within each group.

Without covariates, we found 988 significant (hierarchical False Discovery Rate (FDR) = 5%) associations comprising 85 metabolites and all 29 ASCVD phenotypes available in LIFE-Heart. In LIFE-Adult, we found 539 significant associations comprising all nine ASCVD phenotypes and 92 metabolites (Table S3, Figure S2). When controlling for 15 risk factors and confounders, the number of significant associations is reduced considerably to 468 associations of 29 phenotypes with 81 metabolites in the LIFE-Heart study and to 146 associations of nine phenotypes with 67 metabolites in the LIFE-Adult study (Figure 1). These associations are independent of the 15 additional covariates, including classical CVD risk factors age, sex, BMI, smoking status, type 2 diabetes status, triglycerides and hypertension. Summary statistics of all associations are presented in Table S4.

An overview of significant (hierarchical FDR = 5%) multivariate associations of metabolites with ASCVD phenotypes is shown in Figure 1. A total of six phenotypes were available in both studies allowing replication of results. Criteria chosen for successful replication were significance at hierarchical FDR = 5% and equal direction of effects. Among the 582 replicable associations, we found 62 replicated significant associations involving six ASCVD phenotypes (number of plaques, plaque status, N-terminal prohormone of brain natriuretic peptide (NT-proBNP), carotid intima-media thickness (cIMT), ankle brachial index (ABI) and ABI-based peripheral artery disease status (ABI-PAD)) and 32 metabolites (Table S4 and Figure S3). Among these, the sum of acylcarnitines, acyetylcarnitine (C2) and the ratio of alanine and acetylcarnitine (Q11:Ala/C2) associated with five ASCVD phenotypes, each.

**Figure 2:**
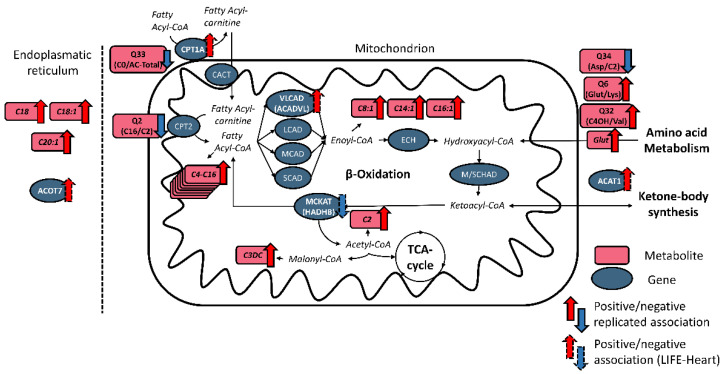
Metabolites (red) and expression of genes (blue) of fatty acid oxidation associated with NT-proBNP in both studies. Replicated (significant after hierarchical FDR = 5% and equal direction of effect) associations are indicated by an arrow showing the direction of effects. Associations only found in LIFE-Heart are indicated by a dashed arrow. Involved pathways comprise reactions in the endoplasmic reticulum or the mitochondrion. For each associating feature, the direction of effect is indicated by an upward (red) or downward (blue) arrow for positive or negative association of the metabolite with NT-proBNP. Most metabolite/gene associations affect mitochondrial β-oxidation. Very long, long- medium- and short-chain acylcarntines accumulate with increasing NT-proBNP, as well as acetylcarnitine (C2) and malonylcarnitine (C3DC). Increase of glutarylcarnitine (Glut) and several ratios (Q6:Glut/Lys, Q32:C4OH/Val, Q34:Asp/C2) indicate increased mobilization of amino acids for intermediates of the β-oxidation. When several enzymes can catalyze a reaction step, the specific gene is given in parentheses. Abbreviations: VLCAD = very-long-chain acyl-CoA dehydrogenase, LCAD = long-chain acyl-CoA dehydrogenase, MCAD = medium-chain acyl-CoA dehydrogenase, SCAD= short-chain acyl-CoA dehydrogenase, CPT1/CPT2 = carnitine palmitoyl transferase type 1/2, CACT = carnitine acylcarnitine translocase, ECH = enoyl-CoA hydratase, M/SCHAD = medium/short-chain hydroxyacyl-CoA dehydrogenase, MCKAT = medium-chain ketoacyl-CoA thiolase, TCA-cylce = tricarbolic cycle, ACADVL = very long chain acyl-CoA dehydrogenase, ACAT1 = acetyl-CoA C-acetyltransferase, HADHB = 3-ketoacyl-CoA thiolase, ACOT7 = acyl-CoA thioesterase 7.

Notably, we observed replicated associations of 37 unique metabolites of all stages of fatty acid oxidation pathways with NT-proBNP, a well-established biomarker of left ventricular dysfunction and heart failure (HF). Replicated associations for metabolites are listed in Table S4. For example, levels of acetylcarnitine (C2) are strongly associated with NT-proBNP (LIFE-Heart: β = 0.14, SE = 0.013, *p* = 3 × 10^−26^; LIFE-Adult: β = 0.11, SE = 0.016, *p* = 1.35 × 10^−11^). Short-, medium-, long- and very long-chain acylcarnitines, malonylcarnitine (C3DC), 3-Hydroxy-butyryl-carnitine (C4OH), glutarylcarnitine (Glut), as well as the amino acids citrulline (Cit), glutamic acid (Glu), glycine (Gly), methylhistidine (MeHis), pipecolic acid (PiPA), tryptophan (Trp) are all consistently positively associated. Pathways of the replicated associations with NT-proBNP are illustrated in Figure 2.

Parameters of coronary angiography and echocardiography were only available in LIFE-Heart. These parameters showed several associations across the metabolome. For example, CAD status (indicating healthy or sclerotic coronary arteries without stenosis vs. patients with significantly sclerotic arteries) was associated with 29 metabolites.

### 2.2. NT-proBNP Associated with Metabolites and Genes Involved in Fatty Acid Metabolism

To complement the identified associations of metabolites with ASCVD phenotypes, we considered blood expression of genes related to fatty acid metabolism. We performed a multivariable association study of gene-expressions from peripheral blood mononuclear cells (PBMCs) in the LIFE-Heart study (see methods). Additionally, we aimed to confirm these associations in whole blood gene expression available in LIFE-Adult. Six phenotypes of peripheral (ABI, ABI-PAD) and carotid ASCVD (mean cIMT, number of plaques and plaque status) and NT-proBNP were considered for this purpose. Considering 145 probes of 91 genes involved in the pathways “fatty acid catabolic process” (GO:0009062) and “cellular ketone body metabolic process” (GO:0046950), we found 18 significant associations, 17 with NT-proBNP and one with ABI-PAD with PBMC gene expression in LIFE-Heart. However, none of these associations could be confirmed for the whole blood gene expression data in LIFE-Adult. Only one significant association with ABI was found. Full association summary statistics are available in Table S9.

We report associations of genes involved in fatty acid oxidation pathways in the peroxisome, the endoplasmatic reticulum, as well as the mitochondrion, indicating changes in a multitude of pathways in association with NT-proBNP. A gene positively associated with NT-proBNP and involved in fatty acid oxidation in the endoplasmatic reticulum was ACOT7, coding for acyl-CoA thioesterase 7, responsible for hydrolyzing the CoA thioester of long-chain fatty acids. NT-proBNP-associated genes involved in peroxisomal fatty acid oxidation comprise the negatively associated ACOX3, coding for the peroxisomal acyl-CoA oxidase, PHYH, coding for phytanoyl-CoA 2-hydroxylase, involved in the α-oxidation of branched-chain fatty acids and HSD17B4, coding for hydroxysteroid 17-beta dehydrogenase 4, a protein involved in peroxisomal fatty acid β-oxidation. Positively associated genes comprise ACSL5, coding for the acyl-CoA synthetase long chain family member 5 and SLC27A2, coding for the solute carrier family 27 member 2, both involved in the conversion of long-chain fatty acids into fatty acyl-CoA esters. Genes involved in mitochondrial β-oxidation that showed upregulation with increased NT-proBNP comprise CPT1A, the liver isoform of the carnitine O-palmitoyltransferase 1, ACADVL, the very long chain acyl-CoA dehydrogenase and ACAT1, coding for the acetyl-CoA C-acetyltransferase, necessary for ketone-body formation and amino acid degradation. One downregulated mitochondrial gene involved in fatty acid oxidation was HADHB, coding for the 3-ketoacyl-CoA thiolase that is necessary for the final step of the β-oxidation. Pathways of association genes involved in mitochondrial β-oxidation are illustrated in Figure 2.

### 2.3. Metabolic Profile as Predictor of Coronary Artery Disease

Since CAD status (significant stenosis as a luminal narrowing of >50%) was multivariably associated with 29 metabolites, we aimed at improving CAD risk prediction beyond classical risk factors using these associated features. Since coronary-angiography is often negative, i.e., no relevant lesions could be detected, it is of clinical importance to improve risk prediction prior to this invasive assessment [15]. In a logistic regression model, the variance of CAD status is explained to a moderate degree (Nagelkerke pseudo-r^2^ = 0.16) by nine classical risk factors (age, sex, log-BMI, hypertension status, smoking status, T2D status, triglycerides, LDL cholesterol and estimated glomerular filtration rate (eGFR)). We fit three models using multivariate penalized logistic regression (see methods): (1) a model only including the nine risk factors, (2) a model including 92 metabolites (excluding five metabolites with ≥10% missing, see methods) and (3) a model combining these predictors. For model development, we split data into a training and a validation set (see methods). In the training data, the area under the receiver operating characteristic (ROC) curve (AUC) of each cross validation (CV) fold are listed in Table S5. Mean AUC of the CV-runs of the risk factors-only model as well as the metabolites-only model was 0.71 and 0.77 for the combined model. Regression weights of all predictors of the combined model fitted to the training data are given in Table S6. Coefficients of the risk factor-only and the metabolites-only model are presented in Tables S7 and S8.

**Figure 3:**
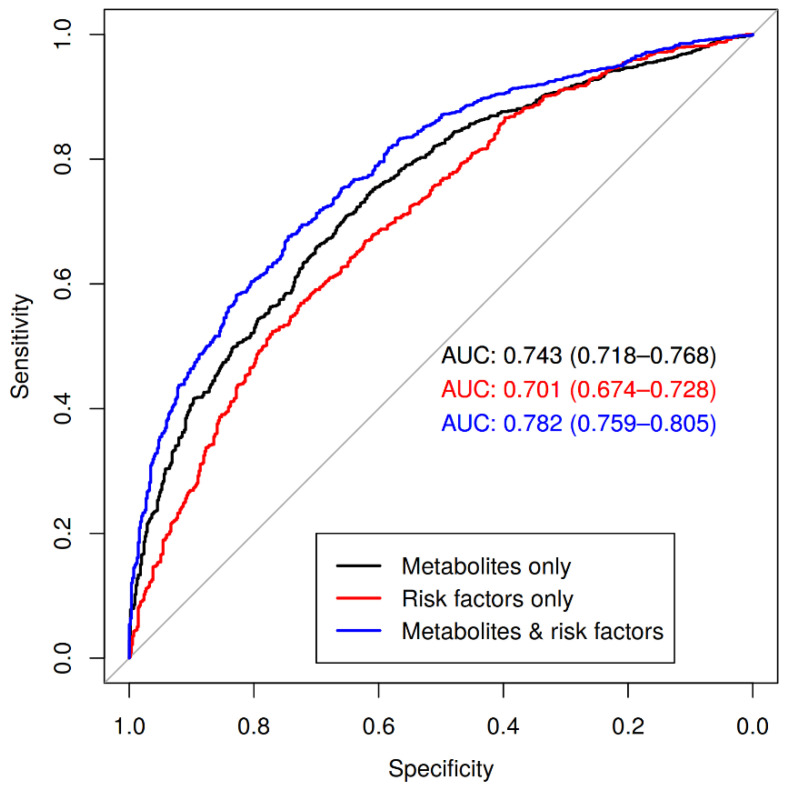
Performance of metabolites and/or risk factors to predict CAD. We present receiver-operating-characteristic (ROC) and respective areas under the ROC curve (AUC) with corresponding 95% confidence intervals. Black: model including only metabolites as predictors; red: model of nine risk factors; blue: model including all risk factors and metabolites as predictors. While the risk factors-only model performs worst, the combined model shows an increase in predictive ability over its competitors (*p* = 3.1 × 10^−18^ for combined vs. risk factors-only, *p* = 1.5 × 10^−4^ for combined vs. metabolites-only, one-sided Delong test). The difference between metabolites-only vs. risk factors-only is also significant (*p* = 0.01795, two-sided Delong test).

For comparing AUC of the three models, we estimated the AUC statistics of each model on the held-back validation data set and performed paired Delong tests. AUCs were 0.7 (95%-CI 0.67–0.73), 0.74 (95%-CI 0.72–0.77) and 0.78 (95%-CI 0.76–0.80) for the risk factor-only, metabolite-only and the combined model, respectively (Figure 3). We detected a small significant difference between the risk factor-only and metabolites-only models in favor of the metabolites-only model (two-sided Delong test for two correlated ROC curves, Z = 2.4, *p*-value = 0.01795). The combined model performed significantly better in the prediction of CAD status than both the metabolites only model (Z =−3.6, *p*-value = 1.5 × 10^−4^) and the risk factors-only model (Z =−8.6, *p*-value = 3.1 × 10^−18^).

## 3. Discussion

### 3.1. Metabolites of Fatty Acid Oxidation Reliably Associate with ASCVD Phenotypes

In this study, we analyzed the relationships between blood metabolites and different traits of ASCVD in two independent cohorts. Several associations, in particular with CAD status and NT-proBNP as a marker of HF were found. Based on these associations, we performed an integrative pathway-based analysis of metabolic genes and their relationships with ASCVD phenotypes available in both studies. Finally, we developed a metabolite-based score to distinguish CAD cases (significant stenosis as a luminal narrowing of >50%) from controls. Metabolites provide a moderate increment in prediction performance compared to classical risk factors. To the best of our knowledge, this is the largest association study of cardiovascular health-associated changes in the metabolome and the first study of the effects of the whole blood metabolome and blood gene expression on a multitude of ASCVD phenotypes including echocardiography, carotid, coronary and peripheral atherosclerosis. We studied two large cohorts allowing replication of the found metabolite associations. Replicated findings suggest changes of metabolites involved in mitochondrial fatty acid and amino acid metabolism. Specifically, we observed associations of a wide range of metabolites, including acylcarnitines of all chain-lengths, with NT-proBNP.

Associations of complex ASCVD phenotypes with metabolite levels are subject to a high degree of confounding. We determined 15 such covariates and included them in our regression models to reduce confounding bias as far as possible. The additional predictors led to an overall reduction in significant associations (at hierarchical FDR = 5%) from 988 (unadjusted) to 468 adjusted associations of 29 phenotypes and 81 metabolites in the LIFE-Heart study and from 539 to 146 associations of nine phenotypes and 67 metabolites in the LIFE-Adult study. The high impact of covariates is also evident in the association results of the models including these 15 covariates as predictors and ASCVD phenotype as outcome. Here, explained variances reached up to r^2^ = 0.46 for brachial-ankle pulse wave velocity with ten significant predictors, highlighting the importance of accounting for covariates to identify spurious associations (Table S10 and Figure S5). Additional confidence in the associations can be acquired by replication in independent studies. 

In total, we report 62 replicated associations of six ASCVD phenotypes and 39 metabolites. The most replicated associations (*n* = 37) were reported for NT-proBNP, an established marker of HF. Confidence of our associations and directions of effect is also strengthened by confirming known effects: for example, the ratio Q2:C16/C2 was negatively associated with NT-proBNP in both of our studies. Ratios of long-chain acylcarnitines such as palmitoylcarnitine (C16), octadecenoylcarnitine (C18:1) and the carnitine conjugate of the end-product of the TCA-cycle acetyl-CoA, acetylcarintine (C2), can be used to diagnose CPT2 deficiency [16]. A low ratio is indicative of an inhibition of the conversion of acylcarnitine to acyl-CoA via the carnitine palmitoyltransferase 2 (CPT2) [17]. Experiments in mice have shown that a CPT2 deficiency in the heart can induce severe heart failure phenotypes [18]. C18:1 was significantly associated with 18 phenotypes across LIFE-Adult or LIFE-Heart, although only the association with NT-proBNP could be found in both studies. The ratio Q3:(C16 + C18:1)/C2 is commonly used to indicate CPT2-deficiency. However, we found no association with any ASCVD phenotype, but Q2:C16/C2 and Q3:(C16 + C18:1)/C2 are highly correlated in LIFE-Adult (Pearson’s ρ = 0.89) and LIFE-Heart (Pearson’s ρ = 0.90).

C2 production buffers acetyl-CoA levels and is important for maintaining metabolic flexibility by reducing the inhibition of the pyruvate dehydrogenase, the rate limiting enzyme for the entry of pyruvate into the TCA-cycle [10]. Prolonged elevation of blood C2 levels can be a signal of metabolic inflexibility and hypoxia caused by heart failure. Accumulation of C2 and numerous chain-lengths of acylcarnitines with increased NT-proBNP levels have been reported [13,19]. We report corresponding associations with several other phenotypes, e.g., we found replicated positive associations of C2 with plaque burden, ABI-PAD and negative associations with ABI, demonstrating a consistent increase in blood C2 across ASCVD phenotypes.

Numerous other studies investigated changes in blood acylcarnitine levels under ASCVD phenotypes [10,11,12,20]. Accumulation of various chain-length ACs have been described as an indicator of incomplete fatty acid oxidation in response to heart failure with reduced ejection fraction [21]. Additionally, hypertrophy, considered a maladaptive reaction to the failing heart, is characterized by a switch from fatty acid oxidation to glycolysis without increase in glucose oxidation, resulting in the uncoupling of glucose uptake and pyruvate oxidation in the cardiomyocytes [22]. We report positive associations of various chain-length acylcarnitines of all steps of the mitochondrial β-oxidation with NT-proBNP: acetylcarnitine (C2), malonylcarnitine (C3DC), butyrylcarnitine (C4), 3-Hydroxy-butyryl-carnitine (C4OH), hexanoylcarnitine (C6), octanoylcarnitine (C8), octenoylcarnitine (C8:1), decanoylcarnitine (C10), dodecanoylcarnitine (C12), myristoylcarnitine (C14), tetradecenoylcarnitine (C14:1), palmitoylcarnitine (C16), hexadecenoylcarnitine (C16:1), stearoylcarnitine (C18), octadecenoylcarnitine (C18:1) and Cis-11-eicosenoic acid (C20:1). This demonstrates the capabilities of metabolomics from dried blood for providing detailed images of the cellular metabolism.

These associations provide a detailed picture of short- (C3–C5), medium- (C6–C12), long- (C16–C20) and very long-chain (>C20) acylcarnitine accumulation. For instance, Malonyl-CoA is formed from carboxylating acetyl-CoA via the acetyl-CoA decarboxylase. The reaction is reversed by the malonyl-CoA decarboxylase. Thus, malonyl-CoA decarboxylase deficiency can lead to malonyl-CoA (and malonylcarnitine) accumulation, which acts as an inhibitor of CPT1 activity, thus limiting mitochondrial β-oxidation [23]. Increased levels of long-chain ACs were also found to be associated with worse clinical outcome in heart failure [10]. Octadecenoylcarnitine (C18:1) showed significant differences in patients with heart failure with reduced ejection fraction vs. controls and heart failure without reduced ejection fraction, in accordance with our observations [21]. Additionally, the sum of total ACs associated with five out of six replicable ASCVD phenotypes (ABI, ABI-PAD, plaque status, number of plaques and NT-proBNP), again highlighting the strong connection between these phenotypes and disturbances in the energy metabolism. Apart from the degradation of fatty acids, dysregulation of other pathways of the energy metabolism, such as the TCA cycle was reported. A decrease in most intermediary metabolites during heart failure was observed in [24,25]. It would be of interest to validate these findings in our data when respective metabolites are measured in the future.

### 3.2. PBMC Expression of Genes Involved in Oxidation of Fatty Acids Associated with NT-proBNP in LIFE-Heart

We analyzed associations of the blood expression of 91 genes involved in the oxidation of fatty acids and the ketone body metabolism to several ASCVD phenotypes. We found 17 associations of genes with NT-proBNP, a well-established biomarker of left ventricular dysfunction, pressure overload and myocardial hypertrophy in LIFE-Heart [26]. None of these associations could be observed in whole blood gene expression in LIFE-Adult. However, considering these specifically selected set of genes that we analyzed, these associations are plausible and warrant further investigation.

CPT1A is the liver isoform of the rate-limiting enzyme of the β-oxidation that conjugates a carnitine group to palmitoyl-CoA for transport across the outer mitochondrial membrane, while ACADVL facilitates the dehydrogenation of palmitoyl-CoA and Myristoyl-CoA in the initial steps of the mitochondrial β-oxidation [27,28]. The increased expression of ACADVL might serve an adaptive role in counteracting the accumulation of toxic lipids. The observed positive association of CPT1A expression and NT-proBNP is consistent with previous reports of a shift in myocardial metabolism to a fetal energy metabolism that prioritizes the less oxygen-demanding glycolysis over palmitate oxidation and is characterized by a lower capacity for mitochondrial oxidative metabolism [23]. CPT1A has a higher affinity to carnitine (C0) and a lower sensitivity to inhibition by malonyl-CoA than the muscle isoform CPT1B, which is predominantly expressed in the heart.

Thus, increased expression of CPT1A may act as a way to maintain fatty acid oxidation at a lower rate under stress conditions in the failing heart, such as a limited oxygen availability, reduced carnitine availability and accumulation of β-oxidation intermediates [29]. This shift away from fatty acid oxidation to glycolysis and the uncoupling from pyruvate oxidation eventually leads to a loss of metabolic flexibility [30,31]. This association further strengthens the assumed contribution of this pathway to the metabolic remodeling occurring during heart failure related due to reduced left-ventricular ejection fraction and pressure overload.

Increased expression of ABHD2, which we observed with higher NT-proBNP levels, was previously observed in human coronary artherosclerotic lesions [32]. The gene encodes a α/β hydrolase fold domain protein with unclear function [33]. The expression was specifically attributed to macrophages and smooth muscle cells and may play a role in macrophage infiltration to atherosclerotic lesions [32]. However, we only observed associations of the gene with NT-proBNP but not with plaque burden.

Failure to confirm associations found in PBMC gene expression in whole blood can have multiple reasons: (1) the difference in tissue, whole blood in LIFE-Adult and PBMCs may lead to different expression profiles to be analyzed; (2) the low incidence of CAD in the cross-sectional LIFE-Adult in comparison to LIFE-Heart, which specifically recruited CAD patients and (3) the associations can be false-positives and changes in the energy metabolism in association with ASCVD phenotypes may not be reflected in blood gene expression levels.

### 3.3. Full Metabolite Profile Is Predictive for Coronary Artery Disease

We leveraged the whole metabolite profile for prediction of significant coronary artery stenosis (CAD status). Due to the invasive nature of this phenotype, it is of potential clinical interest to improve classical risk models using metabolite profiles. Clinical models predicting stenosis typically focus on asymptomatic carotid stenosis (ACS), defined by the degree of luminal narrowing. In our study, we defined the phenotype for significant stenosis as a luminal narrowing of >50%. We demonstrate that a model including blood amino acid and acylcarnitine levels significantly improves performance. However, the gain in AUC is moderate.

The lack of an external validation cohort could result in biased results in model performance despite the sample splitting strategy. Best performing models for detecting significant stenosis (>50%) in the validation cohort in a recent review of ACS prediction models reached up to 0.75 (95%-CI 0.74–0.75) for a model including age, sex, smoking, hypertension, hypercholesterolemia, diabetes mellitus, vascular and cerebrovascular disease, measured blood pressure and blood lipids [15]. The first two prediction models we tested included either only the metabolites or the risk factors. The first prediction model included the classical risk factors age, sex, BMI, smoking status, hypertension status, type 2 diabetes mellitus status, LDL-cholesterol, triglycerides and eGFR, which are commonly used in established clinical prediction scores. This risk factor-only model performed slightly worse than the models discussed in Poorthuis et al. (AUC = 0.7, 95%-CI 0.67–0.73) [15]. The metabolites-only model discriminated slightly better than the risk factor-only model, with an AUC = 0.74 (95%-CI 0.72–0.77) and performed comparable to models proposed in the literature. The combined model significantly improves on both models (AUC = 0.82, 95%-CI 0.79–0.84). Considering the high number of predictors added to the model, the overall gain in predictive ability is moderate.

### 3.4. Limitations

The two cohorts considered in this analysis differ with respect to their recruitment strategy, phenotyping and patient characterization. For example, LIFE-Adult is a population-based study while LIFE-Heart is a mono-centric study of patients with suspected or confirmed CAD. However, for overlapping physical examinations, we used the same devices and standard operating procedures. While metabolite data were extracted from dried blood spots in both studies, gene expression data were extracted from different tissues, namely whole blood in LIFE-Adult and PBMCs in LIFE-Heart, leading to different gene expression profiles with reduced comparability. By limiting the scope of the investigation to acylcarnitines, amino acids and genes involved in mitochondrial β-oxidation, further interesting associations could have been missed involving, for example, other glucose oxidation pathways. Additionally, limiting the investigation to blood metabolites and gene expression could further miss changes in tissues most affected by ASCVD and their associated metabolic remodeling, such as hepatic or myocardial tissues. We failed to confirm associations with gene expression in LIFE-Adult, lowering the confidence in the associations we reported in the LIFE-Heart study. Another limitation is that several ASCVD phenotypes were available in only one of the cohorts, i.e., replication was restricted to overlapping investigations of six phenotypes. In particular this applies to the CAD case/control status. This also limits the confidence in the newly proposed classifier based on classical risk factors and metabolite profiles. External validation of the prediction model in a second cohort would strengthen confidence in the results and reduce bias in the overall performance evaluation.

### 3.5. Conclusions

A growing body of evidence describes a significant role of metabolism in the disease progression and severity of ASCVD through multiple pathways. These range from mal-adaptive to directly causal [22]. We here present a metabolome association study of a large variety of ASCVD phenotypes in two German cohort studies. We report replicated associations of short-, medium-, long- and very long-chain acylcarnitines and amino acids with ASCVD phenotypes, specifically NT-proBNP, supporting a pathomechanistic impact on amino acid and fatty acid metabolism in the mitochondria as well as in the peroxisome. Additionally, we observed associations of expression profiles of genes involved in FAO in relation to NT-proBNP in PBMC gene expression in the LIFE-Heart study. This suggests changes in peroxisomal and mitochondrial fatty acid degradation and a return to a fetal gene expression profile. Finally, we propose a classifier for significant coronary artery stenosis (CAD status) showing moderate improvements compared to a classical risk model. External validation of this risk model is required.

## 4. Materials and Methods

### 4.1. Study Characteristics and Design

We performed single metabolome association studies of several ASCVD phenotypes available in the LIFE-Heart or the LIFE-Adult study. When available in both studies, we conducted a discovery association analysis in LIFE-Heart and a replication analysis in the LIFE-Adult study. An overview of our analysis workflow is shown in Figure S6.

LIFE-Heart: Patients with either suspected or confirmed coronary artery disease (CAD) were recruited at the Heart Center Leipzig, Germany [34]. Confirmed CAD includes cases of stable disease, a history of, as well as acute myocardial infarction (AMI). Since patients were partly recruited after hospital admission, no fasting period was required prior to blood sampling. Metabolite data was available from 5860 participants and matching metabolite and gene expression data was available for 3824 study participants.

LIFE-Adult: The population-based study LIFE-Adult focuses on civilizational diseases and related risk factors of inhabitants of the city of Leipzig, Germany [35]. There, 10,000 sex- and age-stratified randomly selected individuals were recruited. An overnight fast was required for all participants prior to blood sampling. Metabolite data was available from 9646 participants and matching metabolite and gene expression data was available from 3148 participants.

Both studies adhere to the ethical standards of the Declaration of Helsinki and were approved by the ethics committee of the University of Leipzig (LIFE-Heart: Reg. No. 276e2005, LIFE-Adult: Reg. No. 263-2009-14122009). The LIFE-Heart study was registered at ClinicalTrials.gov (accessed on 30 June 2021) (No. NCT00497887). All participants gave their written and informed consent.

We present study characteristics in Table S2. ASCVD phenotypes included parameters of carotid, coronary and peripheral artery disease, vascular stiffness, echocardiographic parameters and NT-proBNP as a biomarker of heart failure.

### 4.2. Metabolite Measurement and Pre-Processing

Sample collection, pretreatment, analysis and quantification of metabolite concentrations are described in detail in Brauer et al. [6]. Metabolite levels were measured from EDTA-whole blood samples that were spotted on filter paper and dried for 3 h. Prior to analysis, samples were stored at −80 °C. For spectrometric analysis, 3 mm punched-out blood spots, corresponding to 3 µL of blood, were extracted using methanol containing isotope-labeled internal standards [6]. After butylation, samples were analyzed batch-wise by flow-injection analysis in an API 2000 or API 4000 tandem mass spectrometer (SCIEX, Darmstadt, Germany). Each 96-well plate contained two quality control samples for the estimation of the inter-assay coefficient of variation [5,7]. ChemoView 1.4.2 (Applied Biosystems, Darmstadt, Germany) software was used to derive absolute metabolite concentrations of 62 metabolites (27 amino acids, 34 acylcarnitines and free carnitine) from concentrations of spiked isotope-labeled internal standards. We calculated the biologically relevant sum of acylcarnitines (total ACs) and 34 ratios of metabolites (denoted as “Q”, followed by a number) that represent reaction equilibria within relevant physiological pathways involving these metabolites. Table S1 provides an overview of the 97 measured analytes and quotient definitions and annotated pathways.

We performed study-wise pre-processing of metabolite data using a previously described workflow [36]. In brief, outliers of +5 × SD of log-transformed data were removed, temporarily excluding observations with a value of zero for this step. Values at zero resulted from measurements below the detection limit, which occured for several metabolites (up to 82% and 84% for 3-hydroxy-octadecanoylcarnitine (C18OH)). The percentage of observations with zero-values is shown in Table S1 for each metabolite. In a previous study, we investigated the impact of zero inflation on association statistics and found an increased rate of false negatives, but not false positive associations in the affected metabolites [37]. Our 5 × SD filter removed at most 0.3% of measurements of any metabolite in any study. Subsequently, we inverse-normal transformed all metabolites. Finally, we removed technical batch-effects caused by plate numbers using an empirical Bayes method as implemented in the ‘ComBat’ function of the ‘sva’ R package [38,39].

### 4.3. Gene-Expression Measurement and Pre-Processing

RNA of 4354 patients of the LIFE-Heart study was extracted from peripheral blood mononuclear cells (PBMCs). In LIFE-Adult, whole blood samples of 3173 participants were collected for RNA extraction. Illumina HT-12 v4 Expression BeadChips (Illumina, San Diego, CA, USA) were used for RNA measurement. Scanning on the Illumina iScan was performed according to the manufacturer’s specifications. See Burkhardt et al. and Holdt et al. for a complete description of the sampling and measurement process [40,41].

Pre-processing of gene expression data is described in detail in Kirsten et al. [42]. The workflow uses ‘Bioconductor’ functionality and is implemented in the ‘HT12ProcessoR’ R package (https://github.com/holgerman/HT12ProcessoR (accessed on 30 June 2021)) [43]. Whole blood and PBMC gene expression profiles of the two studies were pre-processed separately. For this, log_2_-transformation of data was followed by quantile-normalization. Probes expressed in less than 5% of all samples were also removed. Samples with an atypical number (±3 × IQR from the median) of expressed genes were excluded. Probes designed for quality analysis were used to calculate a Mahalanobis distance of samples. Outliers of ±3 × IQR from the median were considered as samples with atypical quality and were removed. Technical batch effects as indicated by the Sentrix barcode were removed using the ‘ComBat’ empirical Bayes method [39]. Probes still associating with batches after Bonferroni correction were removed from further analyses. We removed samples with a Euclidean distance of expression values > 4 × IQR from the median as outliers. The ‘Ingenuity Pathway Analysis’ database (QIAGEN Inc., Venlo, Netherlands) was used to map probes to unique genes.

### 4.4. Analysis of Cofactors

We analyzed the impact of 31 potential covariates on metabolite levels in both studies using the workflow implemented in our pipeline ‘Metabolite Investigator’ [36]. Variables are considered relevant if their partial explained variance is larger than 1% for at least one metabolite in one study in a multivariate model containing all other covariates as predictors. All available potential covariates are shown in Table S2. Smoking status was defined as never/former smoker vs. current smokers. The diabetes type 2 status was defined as either Hba1c > 6.5%, anamnestic history of diabetes or application of diabetes-specific medication, defined as Anatomical Therapeutic Chemical (ATC) classification category ATC A10. The variables BMI and NT-proBNP exhibited a heavily skewed distribution and were log-transformed prior to analysis. We scaled all continuous covariates to a mean of µ = 0 and a standard deviation σ = 1.

The effects of lipid modifying agents and hypertension were force-included into the model because of their known effect on blood metabolites as well as ASCVD phenotypes. The variables selected as covariates for our metabolome association analyses were age, sex, BMI, smoking status, hours fasted, hypertension status, type 2 diabetes mellitus status, lipid modifying agents (ATC C10), sex hormones or modulators of the reproductive system (ATC G03), high-density lipoprotein (HDL) cholesterol, triglyceride, hematocrit, leucocytes, lymphocyte and monocyte percentage (Figure S1). We present the partial explained variances of these variables per ASCVD phenotype in Figure S5 and Table S10.

### 4.5. Discovery and Replication of Metabolite Associations with ASCVD Phenotypes

We calculated associations of blood metabolites with 31 ASCVD phenotypes separately in LIFE-Heart (discovery) and LIFE-Adult (replication) with the metabolites as the response variable and the ASCVD phenotypes available in both studies and selected covariates as predictor variables. Phenotypes available in both studies comprise NT-proBNP, mean of the left and right carotid intima-media thickness (cIMT), a score indicating the sum of plaques at the Arteria carotis communis and bulbus (plaque score) and the ankle brachial index (ABI), measured according to the American Heart Association’s specifications [44]. Peripheral artery disease (ABI-PAD) was defined as ABI < 0.9. We also analyzed the case/control status of any plaque at arteria carotis communis or bulbus. Data on pulse wave velocity were only available in LIFE-Adult. Echocardiography and coronary angiography results were only available in LIFE-Heart. Thus, we only performed discovery analyses of these traits without replication in another study. Available data in one or both studies are shown in Table S2.

We conducted all analyses using R 3.6.0 [45]. We fitted generalized linear models with a logit-link for binary phenotypes (‘glm’ function of the ‘stats’ package using the ‘family = binomial(link = ”logit”)’ parameter) and a multivariate linear model for continuous phenotypes (‘lm’ function of the ‘stats’ R-package). We calculated the Nagelkerke pseudo-R^2^ as an indicator of the explained variance for binary phenotypes [46]. For metabolite associations, we used metabolites as the response variable and ASCVD phenotypes and covariates as predictor variables and for the associations of the ASCVD phenotypes with the confounders and risk factors, we used the ASCVD phenotypes as response variables and confounders and risk factors as predictors.

We applied a hierarchical, per-family multiple testing correction of *p*-values. For this, *p*-values were first corrected on the single metabolite level (local adjustment), followed by adjustment across metabolites (global adjustment) [47,48]. We controlled the false discovery rate (FDR) on both levels at FDR = 5% using the Benjamini–Hochberg procedure implemented in the ‘*p*.adjust’ function using ‘method = ”BH”’ as parameter [49].

### 4.6. Discovery and Replication of Associations of ASCVD Phenotypes with Blood Gene Expression

To support our metabolite associations, we investigated associations of selected gene expression probes with the six ASCVD-phenotypes available in both studies. For this, we analyzed a subset of genes contained in the GO-terms “fatty acid catabolic process” (GO:0009062) and “cellular ketone body metabolic process” (GO:0046950), accessed on 7 July 2021 [50,51]. These lists included 98 and eight genes, of which 90 and one gene, a total of 91 genes, could be mapped to gene expression probes available in both LIFE-Adult and LIFE-Heart. We calculated associations with ASCVD phenotypes using the ‘lmFit’ and ‘eBayes’ functions of the ‘limma’ Bioconductor package [52]. We fit multivariate regression models with the gene expression probe as the response variable and one ASCVD phenotype each and all 15 covariates as predictor variables separately in each study. We applied hierarchical correction for multiple testing using the ASCVD phenotype as the family for local adjustment and the genes for global correction [48]. Summary statistics of all associations are presented in Table S9.

### 4.7. Classification/Risk-Prediction Analysis

The workflow for model development, validation and comparison is illustrated in Figure S8. We aimed to assess the discriminatory ability of the complete metabolite profile to distinguish CAD cases, defined as luminal narrowing of >50% observed during coronary angiography, and respective controls in LIFE-Heart. The predictor is the inverse logit function of a linear function. We compare three different models: (1) a model including nine established classical risk factors available in LIFE-Heart (age, sex, BMI, smoking status, T2D status, hypertension status, triglycerides, LDL cholesterol and estimated glomerular filtration rate); (2) a model including only the here considered metabolites achieving a missing rate < 10%, resulting in a total of 92 predictors (excluding Asn, Met, Q22, Q23, and Q24); (3) a model including both the risk factors and these metabolites resulting in 101 predictors. Complete data for all predictors were available for *n* = 4717 samples. We developed a linear predictor based on a multivariate logistic regression model. For development and reporting of the predictor, we adhered to the recommendations of the TRIPOD statement [53].

Due to the high number of predictors in the models including metabolites, we applied a regularizing shrinkage “Ridge Regression” modeling approach [54]. A global shrinkage parameter lambda was determined to reduce the extent of overfitting. A higher lambda results in higher shrinkage of effect estimates and reduced overfitting. The inverse logit function (logistic function) was applied to the predictor to obtain a probability of significant stenosis for each sample.

Model validation and calibration: The data were randomly split into two-thirds (*n* = 3145) for model development and validation and one third (*n* = 1572) for model comparison. Discriminatory abilities of the models were assessed by 10-fold cross validation using the area under the curve (AUC) of the receiver operating characteristics (ROCs) within the validation set. We used the R-packages ‘caret’ and ‘glmnet’ for resampling and model fitting [55,56]. The shrinkage parameter lambda for the model of each of the 10 CV-runs was determined using 200 optimism bootstrap samples [57]. The AUC estimate of each CV-run was averaged for a final assessment of the model’s discriminatory ability within the training data. Subsequently, the model of each scenario was fit on the complete two-thirds of the training data to estimate the final regression weights of each model. For this, the optimal shrinkage parameter lambda was again determined using 200 bootstrap samples and set to λ = 0.01 for the combined model.

Based on model weights and shrinkage parameters estimated from the training data, we compared the performances of the three models in the held-back data (one-third of the data). Model AUCs were computed and tested for difference using DeLong’s test from the R-package ‘pROC’ [58,59]. We tested one-sided for increase in AUC when using the combined model compared to the metabolites only or the risk factors only model and two-sided when comparing the metabolites-only model vs. the risk-factors-only model. We assessed the calibration of the model via the calibration curve showing the relationship between observed and predicted CAD-status using the package ‘CalibrationCurves’ [60,61]. We estimated the intercept and slope of the calibration curve for the combined model on the validation data as β_0_ = 0 and β_1_ = 1.1. The targets for the intercept of β_0_ = 0 and β_1_ = 1 were nearly met, showing overall good calibration of the model, with slight underestimation of risks (Figure S7).

## Data Availability

The data presented in this study are available in the Supplemental Tables.

## Supplementary Materials

The following supporting information can be downloaded at: https://www.mdpi.com/article/10.3390/metabo12030216/s1, Supplemental Figures S1–S8 and Supplemental Tables S1–S10.
