# Supplementary material for: Whole Blood Metabolite Profiles Reflect Changes in Energy Metabolism in Heart Failure
Source: Metabolites. 2022 Feb 27;12(3):216. doi: 10.3390/metabo12030216 (PMC8949022; doi:10.3390/metabo12030216)
Supplement: Supplementary file 1 [file metabolites-12-00216-s001.zip › Supplemental Figures.pdf]

Whole blood metabolite profiles reflect changes in energy metabolism in heart failure – Supplemental Figures

Content

Figure S1 2

Figure S2 3

Figure S3 4

Figure S4 5

Figure S5 6

Figure S6 7

Figure S7 8

Figure S8 9

Figure S1

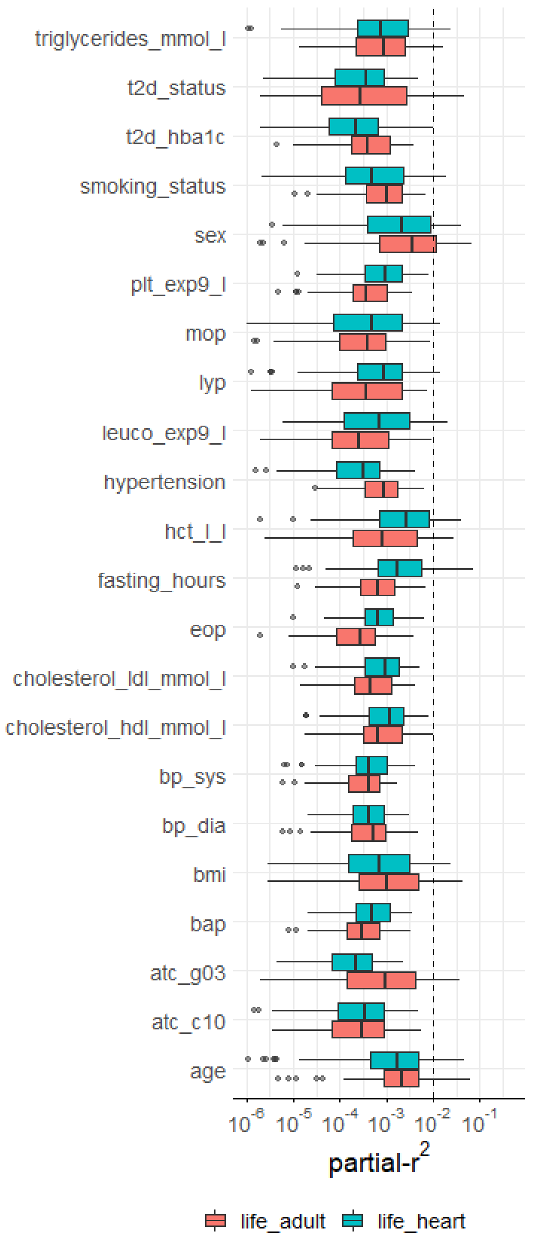

Figure S1. Distributions of 97 metabolites' variances as partial explained by 22 potential confounders in the LIFE-Adult and LIFE-Heart studies. Metabolites' variances explained by a factor on the y-axis must exceed partial-r²>1% (dashed line) to be considered covariates for subsequent association analysis of ASCVD phenotypes and metabolites. Lipid-modifying agents („atc_c10") and hypertension status („hypertension") were force-included because of their known effect on ASCVD phenotypes.

Figure S2

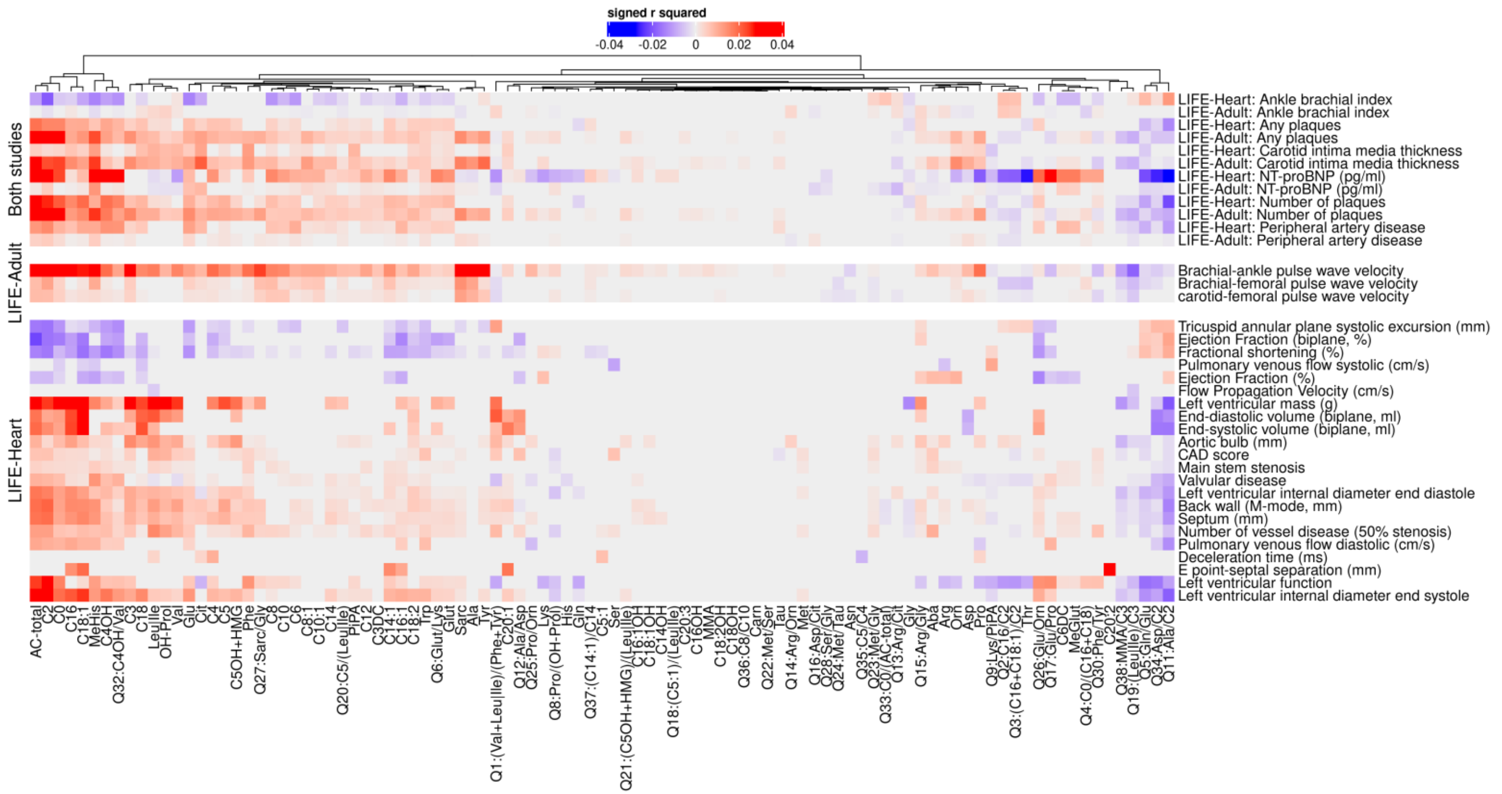

Figure S2. Associations of whole blood metabolites with ASCVD phenotypes without adjustment for covariates in LIFE-Adult and LIFE-Heart. Partial explained variance (r^2) and direction of effects are displayed for each association. Phenotypes are presented in three groups: "Both studies" comprises phenotypes available in both studies, while the other groups are study-specific. Hierarchical clustering based on the Euclidean distance using the complete linkage method was applied for metabolites and phenotypes within each group. Only significant associations are colored.

Figure S3

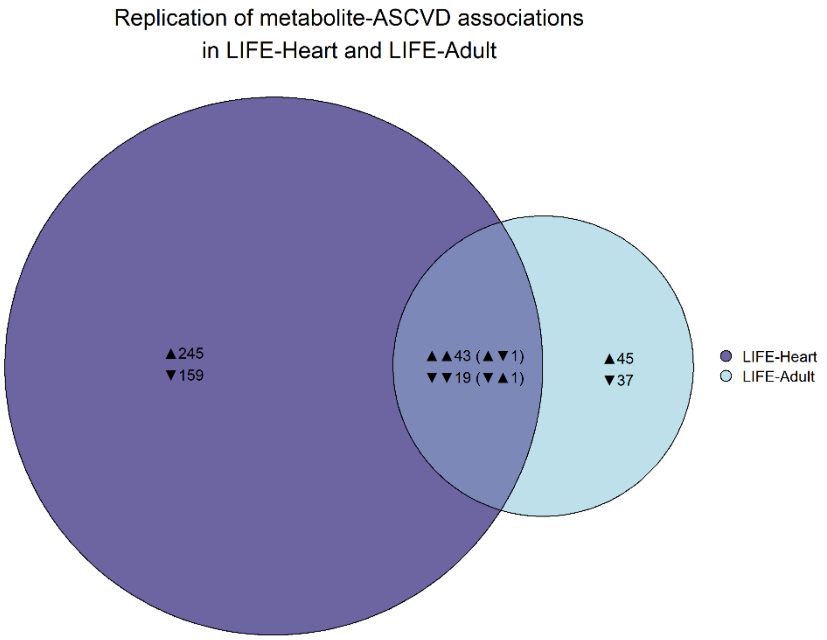

Figure S3. Euler diagram of associations of 97 metabolites with 31 ASCVD phenotypes in LIFE-Heart and LIFE-Adult. The circles represent the total significant associations in each study (hierarchical FDR=5%), with the black triangles representing positive (upwards-facing triangle) and negative (downwards facing triangle) associations. The overlap between the circles represents replicated associations with equal (two upwards- or downwards-facing triangles) or unequal direction (two triangles facing opposite directions). We report 62 replicated associations: 43 with positive effect estimates and 19 with negative effect estimates, while associations with unequal directions of effects were not considered as replicated.

Figure S4

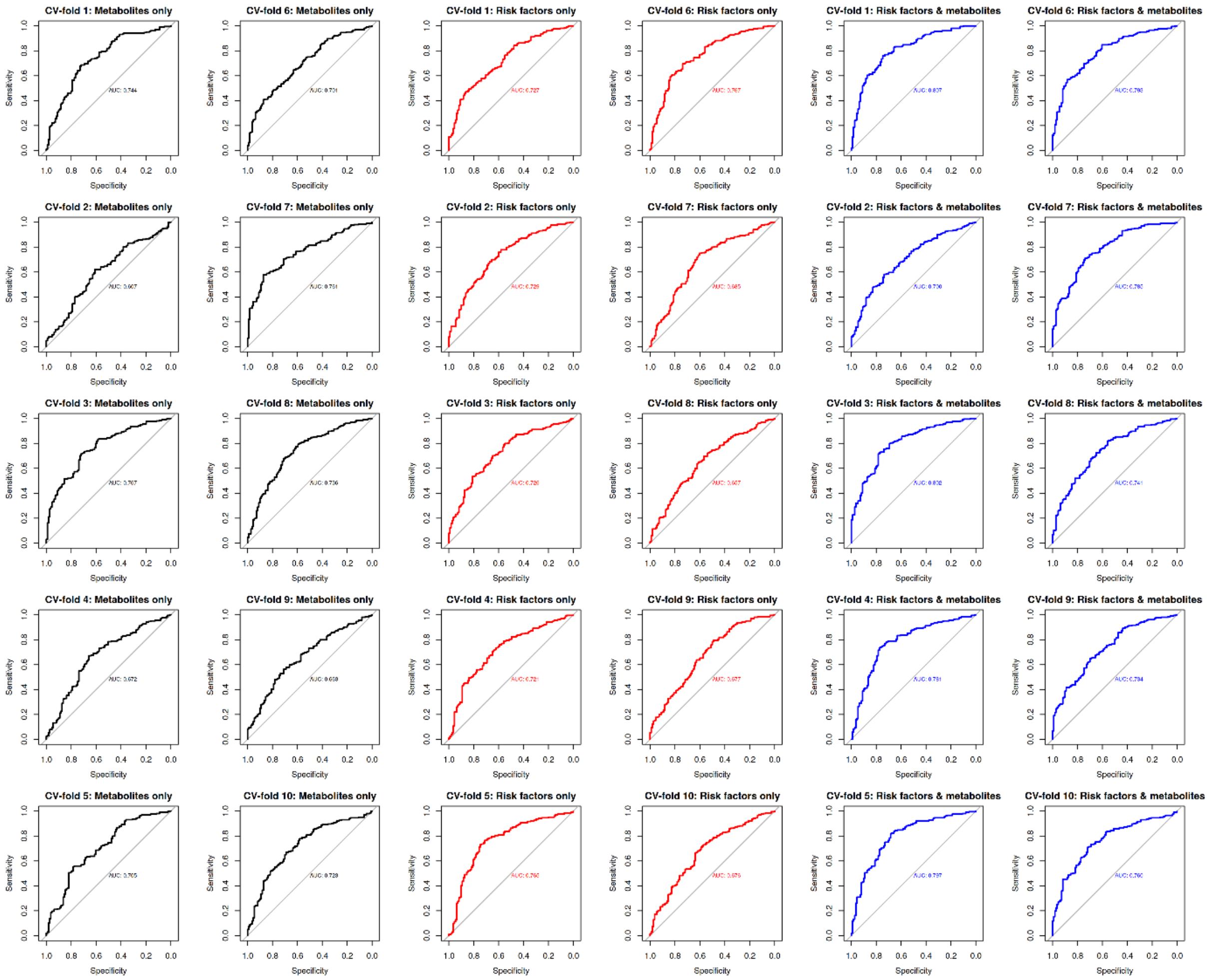

Figure S4. Performance of metabolites and/or risk factors to predict CAD in LIFE-Heart. We present receiver-operating-characteristics (ROC) and respective areas under the curve (AUC). Each image represents the performance of the respective models in each of the cross validation (CV)-runs in the training and validation data (N=3,145). Black: model including only metabolites as predictors; Red: model of nine risk factors; Blue: model including all risk factors and metabolites as predictors. Each image represents results from one cross-validation run in the training data set.

Figure S5

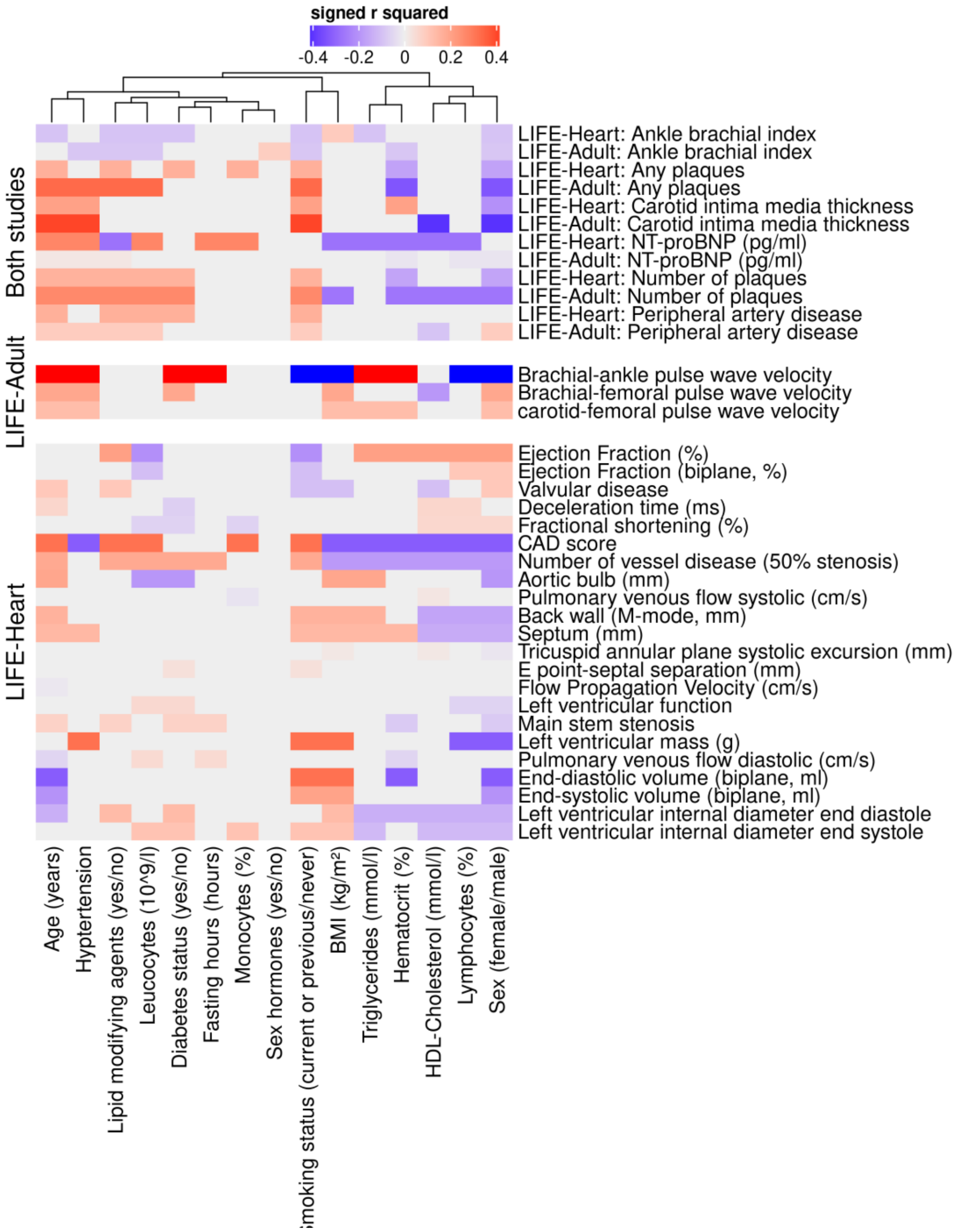

Figure S5. Associations of whole ASCVD phenotypes with 15 confounders and risk factors from multivariable linear regression models in LIFE-Adult and LIFE-Heart selected in the confounder analysis. We used each ASCVD phenotype as single response variable (y-axis) and all 15 confounders and risk factors (x-axis) as joint predictors. Partial explained variance (r^2) and direction of effects are displayed for each association. Phenotypes are presented in three groups: "Both studies" comprises phenotypes available in both studies, while the other groups are study-specific. Hierarchical clustering based on the Euclidean distance using the complete linkage method was applied for metabolites and phenotypes within each group. Only significant associations are colored.

Figure S6

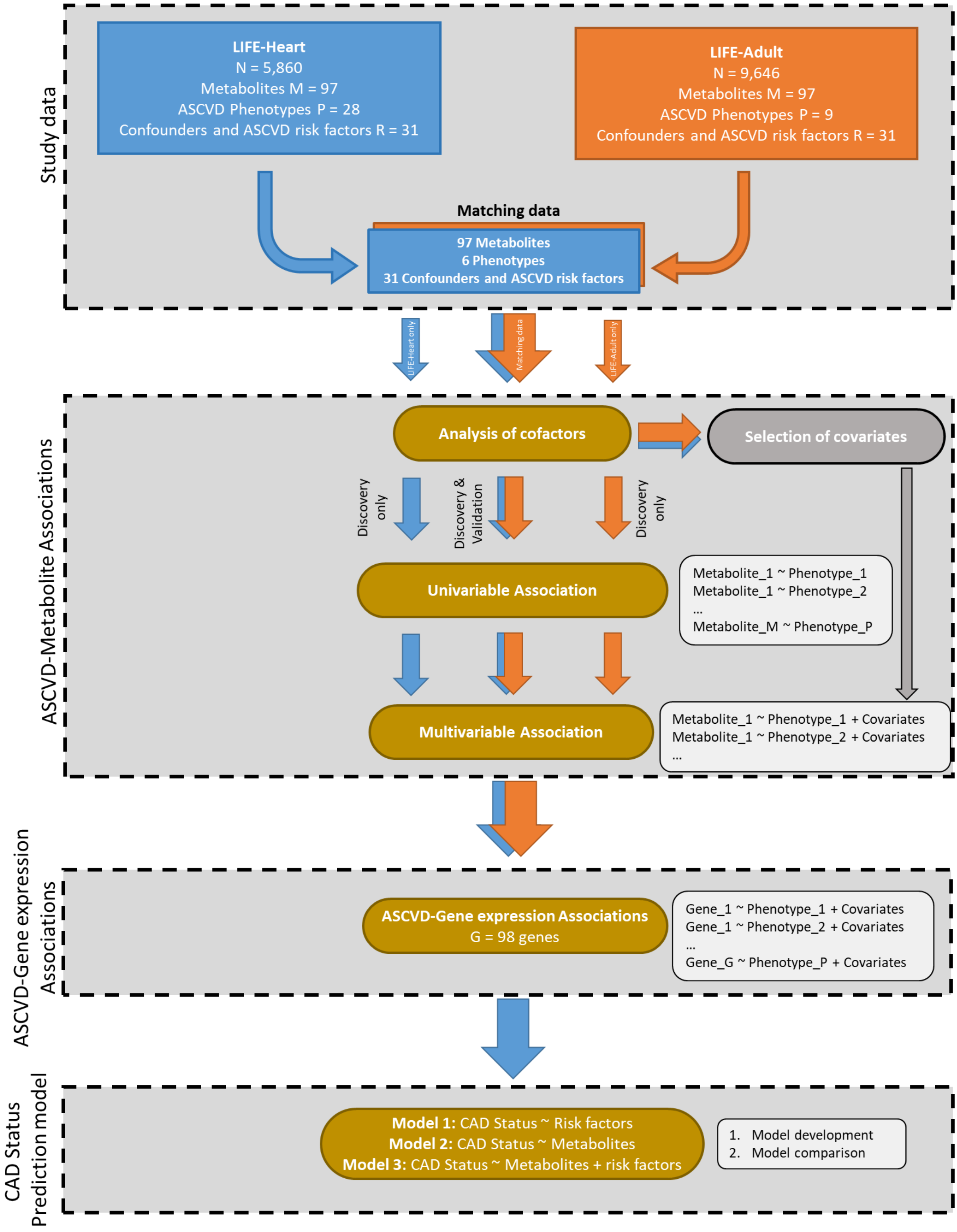

Figure S6. Overview of the analysis workflow employed in this study, omitting data pre-processing. For the association studies, we used matching data available in both the LIFE-Heart and the LIFE-Adult study (six phenotypes) to replicate associations. In the metabolite association analysis, we analyzed all available atherosclerosis and cardiovascular disease (ASCVD) phenotypes. In the gene expression analysis, we analyzed only the six overlapping phenotypes. Due to availability, we fit the model for prediction of coronary artery disease (CAD) on LIFE-Heart data only.

Figure S7

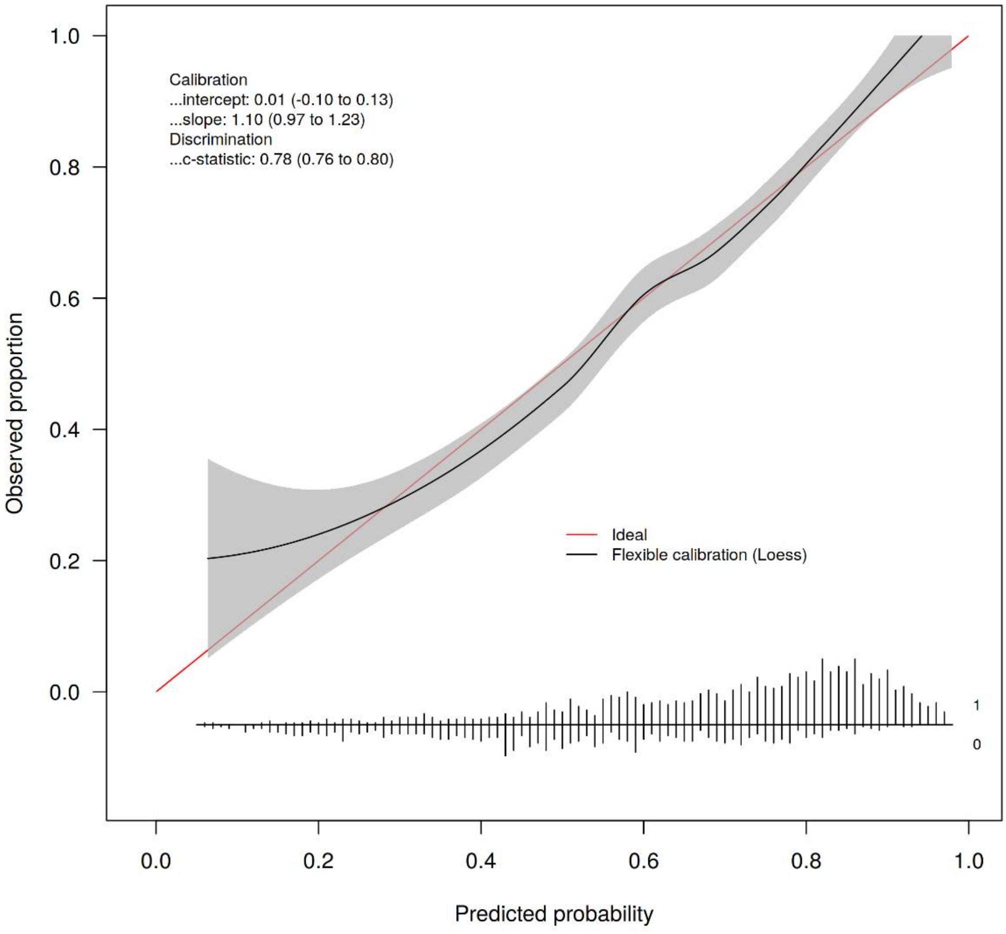

Figure S7. Calibration plot of a model to predict CAD in patients of the LIFE-Heart showing the relationship between the observed proportion of CAD in the test data and the predicted risk. The model incorporates nine classical risk factors and 92 metabolites. The model was fit on 2/3 of available data (training and validation data) and then used to predict CAD in 1/3 of the remaining data (held-back training data). The receiver-operating-characteristics (ROC) and respective areas under the curve (AUC) and 95% confidence interval of the model as well as calibration intercept and slope are given. A calibration intercept near 0 and a slope near 1 indicate overall good calibration, i.e. no over- or under-estimation of risks in the testing data.

Figure S8

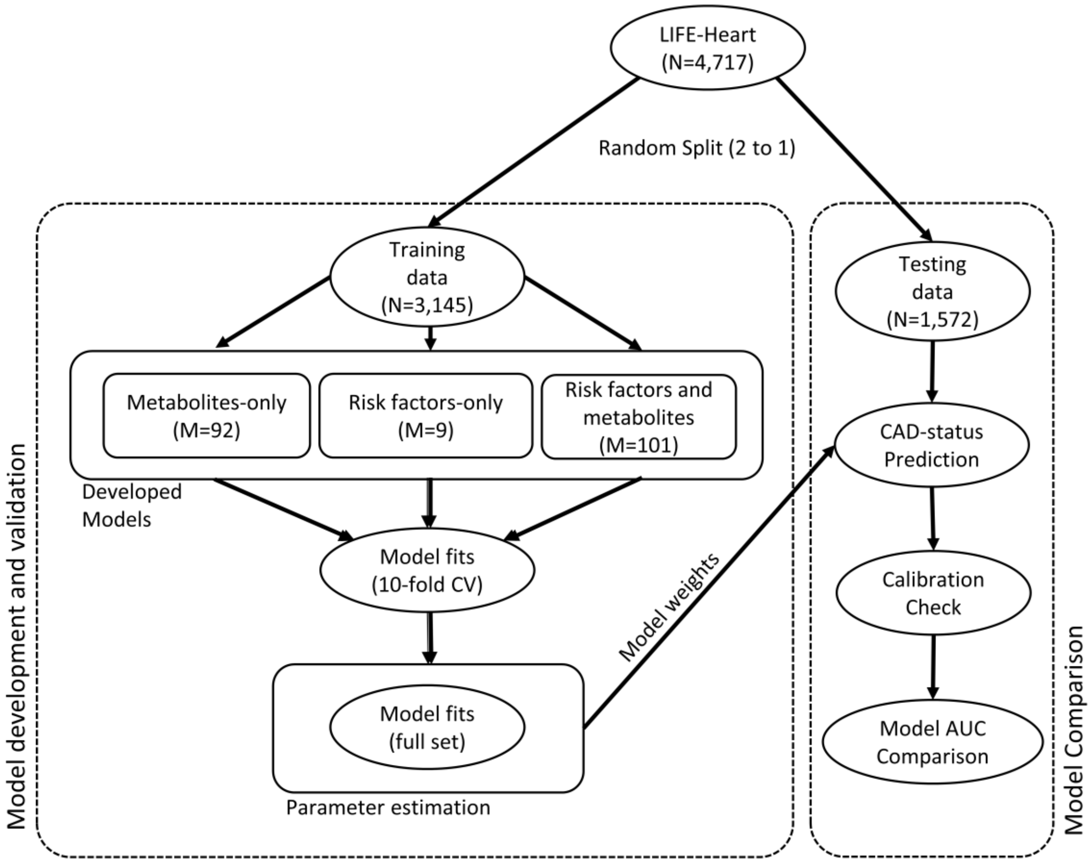

Figure S8. Flow-diagram of the workflow deployed for developing a model to predict CAD in participants of the LIFE-Heart study. The available data (N=4,717) was split into training and validation data (N=3,145) and testing data (N=1,572). Three models were considered in the analysis: 1) a model employing the full metabolite profile, only excluding metabolites with a missing rate <10% (M=92), 2) a model using nine classical risk factors (M=9) and 3) a model combining the risk factors and the metabolite profile for a total of M=101 predictors. The models were fit using a shrinkage modeling approach, employing linear ridge regression models. We evaluated overall model performance using 10-fold cross validation (CV), while estimating the shrinkage parameter for each model using 200 optimism bootstraps in each of the CV-runs. Afterwards, the models were fit on the full training data, again estimating the shrinkage parameter using 200 optimism bootstraps. These models were used to predict CAD in the held-back testing data. We assessed the overall calibration of the models by estimating the calibration intercept and slope of the calibration curve. Afterwards, we compared the model performances by testing for difference in the area under the receiver-operating-characteristic (ROC) curve (AUC) of each model using paired DeLong tests.
